# Outcomes of an Interventional Approach in STEMI and NSTEMI All‐Comer Octo and Nonagenarians

**DOI:** 10.1002/clc.70463

**Published:** 2026-09-09

**Authors:** Charlotte E. P. Siegers, Quinten P. Hoogervorst, Jan van Ramshorst, Sanneke P.M. de Boer, Maurits Dirksen, Victor A. W. M. Umans

**Affiliations:** ^1^ Department of Cardiology Noordwest Ziekenhuisgroep Alkmaar the Netherlands

**Keywords:** ACS, nonagenarians, NSTEMI, octogenarians, PCI, STEMI

## Abstract

**Introduction:**

The number of octogenarians and nonagenarians presenting with (NSTEMI) is rising. However, they are underrepresented in studies. Therefore, we aim to clarify patient characteristics and compare all‐cause mortality of an initial invasive strategy versus optimal medical treatment in (N)STEMI octo and nonagenarians.

**Methods:**

All consecutive patients with (N)STEMI admitted from 2020 until 2023 were included. Patients were divided into invasive and OMT groups. Multi‐variation corrected analyses were performed in two age groups.

**Results:**

775 consecutive patients > 80 years old were analyzed (81 > 90 years). A predominance of women, lower hemoglobin levels, eGFR, and lower rates of coronary angiography and PCI were observed in the nonagenarians. Six patients underwent CABG; all were in the 80–90‐year age group. No differences existed in clinical complication rates between the groups. Invasive treatment demonstrated lower 1 year mortality, predominantly seen in NSTEMI elderly patients.

**Conclusion:**

In octo and nonagenarian (N)STEMI patients, stratifying to an invasive strategy with PCI/CABG appears to be feasible and is associated with improved mortality outcomes. This benefit is predominantly observed in patients with NSTEMI. Stratification can be done immediately on common clinical variables and appropriate shared decision‐making.

## Graphical abstract

All‐cause mortality of an initial invasive strategy versus optimal medical treatment is assessed in 775 all‐comer consecutive (N)STEMI octo/nonagenarians. Nonagenarians were predominantly women, had lower hemoglobin levels, and eGFR. An invasive strategy treatment demonstrated lower 1 year mortality in octo/nonagenarian (N)STEMI patients, most prominently in NSTEMI elderly.

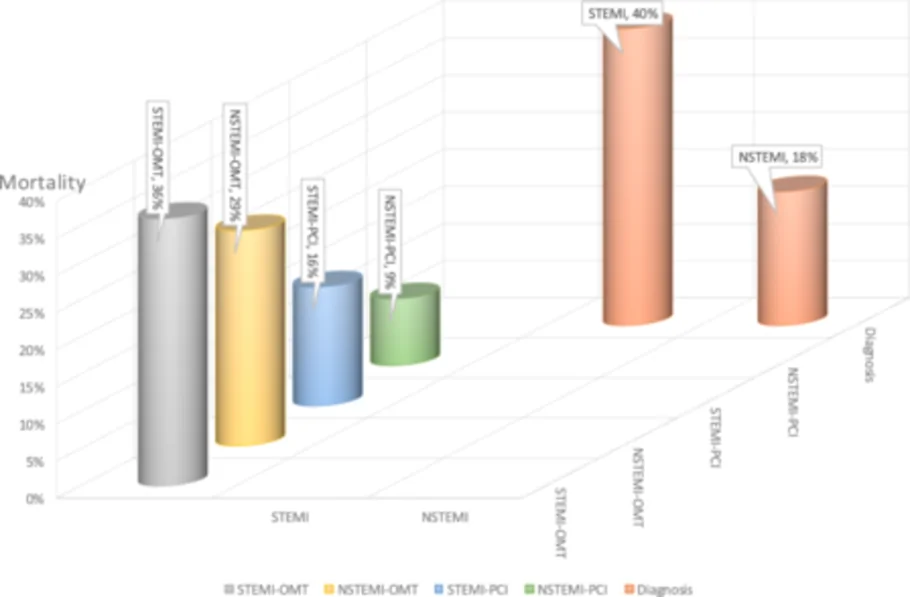

## 1 Introduction

Increasing life expectancy in the Western world has been accompanied by a rising incidence of acute coronary syndrome (ACS) among octogenarians and nonagenarians [1]. ACS is common in the elderly and has a major impact on morbidity, mortality, and quality of life [2]. Treating ACS in octogenarians and nonagenarians is challenging due to multimorbidity, frailty, atypical presentation, and lack of evidence‐based treatment [2].

However, octo and nonagenarians are underrepresented in clinical studies; only a limited number of small studies investigated mortality outcomes following invasive versus conservative treatment strategies [3, 4, 5, 6, 7, 8]. Therefore, current ESC ACS guidelines recommend a holistic strategy for the “very elderly NSTEMI and ACS patient” due to a lack of robust evidence. For patients presenting with STEMI, current guidelines recommend primary PCI when feasible.

Disease management in octo and nonagenarian patients is of major importance when selecting optimal therapeutic strategies. This is particularly true—and challenging—in elderly patients presenting with STEMI, in whom rapid decision‐making is crucial to achieve the potential benefits of invasive treatment.

Since optimal care extends beyond treatment of the underlying pathology it requires a tailored approach that considers patients' individual needs, values, and vulnerabilities [9]. However, trial‐derived evidence may be biased due to the exclusion of the frailest, oldest, and multimorbid patients, which limits generalizability [10].

In this study, we evaluated clinical characteristics and outcomes of consecutive, real‐world, all‐comer octo and nonagenarian patients presenting with (N)STEMI at a regional high‐volume teaching PCI center. Outcomes of invasive versus optimal medical therapy were analyzed, with stratification by age, ACS subtype, and sex.

## 2 Methods

### 2.1 Data Extraction

During the period 2020–2023, all consecutive patients experiencing ST‐elevated myocardial infarction (STEMI) and non‐ ST‐elevated myocardial infarction (NSTEMI) admitted at the Northwest hospital group (NWZ) were included in the EHR database. Approval was given by the local ethics commission. Data were collected from the electronic health record (EHR) using automated extraction scripts to obtain physician‐entered clinical data for patients aged ≥ 80 years with a diagnosis of STEMI or NSTEMI.

The term (N)STEMI is used when outcomes and analyses for the total population of STEMI and NSTEMI patients together are reported.

Information on medical (cardiac) history and risk factors was available in predefined EHR fields for the majority of patients. In cases of missing data, supplementary information was manually retrieved from the EHR and added to the study database.

STEMI was defined as elevated Troponin I, anginal symptoms, and new ST‐segment elevation or left bundle branch block on ECG. NSTEMI was defined as elevated Troponin I in the absence of ST‐segment elevation in the setting of anginal symptoms, including types 1 and 2.

Smoking was defined as current tobacco use. Hypertension was considered present if documented or if systolic blood pressure was ≥ 130 mmHg or diastolic blood pressure ≥ 80 mmHg. Hypercholesterolemia was considered present if reported in the medical history or if total cholesterol was ≥ 6.5 mmol/L or LDL cholesterol ≥ 4.9 mmol/L. Diabetes mellitus was defined as a documented diagnosis of type 1 or type 2 diabetes or fasting plasma glucose ≥ 6.9 mmol/L or HbA1c ≥ 53 mmol/mol.

Strategy decisions were made considering the following considerations: in compliance with the ESC guideline [3] STEMI patients were strategized to an invasive strategy unless known cognitive disabilities, severely decompensated clinical situation or a previous decision to not intervene. All STEMI patient ECG 's are pre‐hospital routinely sent by ambulance nurses to the interventional cardiologist with a follow‐up telephone contact before landing at the catheterization laboratory.

In NSTEMI patients, stratification was consistent with the ESC guideline [3], but given the elderly population, more time was taken to properly consider interventions. Line in STEMI cases, known cognitive disabilities, severely decompensated clinical situations, or a previous decision not to intervene. Secondly, severe renal and hematologic dysfunctions and the outcomes of shared decision‐making were considered.

The primary outcome was all‐cause mortality at 365 days and survival using Cox proportional hazards regression. Percutaneous Coronary Intervention (PCI) was defined as any attempted percutaneous coronary intervention, whether successful or unsuccessful, performed during the index admission. Coronary Artery Bypass Grafting (CABG) was defined as coronary artery bypass grafting performed within 30 days after admission, without a preceding PCI. In‐hospital mortality was ascertained through documentation in the electronic health record (EHR), whereas post‐discharge mortality was verified using municipal registry data.

Bleeding Academic Research Consortium (BARC) type 3a bleeding was defined based on a documented decline in hemoglobin (Hb) levels recorded in the electronic health record (EHR). The Hb decrease was calculated as the difference between the level at admission and the lowest value recorded during hospitalization. A decrease of ≥ 1.86 mmol/L was used as the threshold to meet the BARC 3a bleeding criteria [11].

Cerebrovascular events were identified from the EHR based on diagnoses made by neurologists.

Admission to the intensive care unit (ICU) after initial hospital admission was used as a surrogate marker for post‐infarction or post‐interventional hemodynamic instability.

### 2.2 Statistical Analysis

Statistical analyses were performed using SPSS Statistics version 28 (IBM Corp., Armonk, NY, USA). Continuous variables are presented as mean ± standard deviation (SD) when normally distributed, and as median with interquartile range (25th–75th percentiles) when not normally distributed. Normality was assessed using the Shapiro–Wilk test. Variables with a non‐normal distribution were log‐transformed; if normality was not achieved after transformation, non‐parametric tests were applied. Using the Mann–Whitney U test for two groups and the Kruskal–Wallis test for three groups.

Dichotomic variables are reported as absolute values and as percentages. All tests are two‐sided, and a *p* < 0,05 was considered statistically significant. Chi‐square tests were used for dichotomic outcomes when the assumptions were met. Otherwise, Fisher's exact test was used. Cox regression analyses were used for survival analyses.

Adjustment was performed for the following variables: sex; history of myocardial infarction; prior PCI; prior CABG; cardiovascular risk factors, including smoking, hypertension, hypercholesterolemia, and diabetes mellitus; ACS subtype; lowest hemoglobin level; highest estimated glomerular filtration rate (eGFR); highest C‐reactive protein (CRP) level; and highest troponin level.

Patients were stratified into different groups. In the first analysis, patients were divided into two age‐based groups: 80–89 years and ≥ 90 years. In the second analysis, patients were categorized into four groups based on treatment strategy—optimal medical therapy (OMT) or invasive therapy (PCI or CABG)—within each age group (80–89 years and ≥ 90 years).

Crude and adjusted regression analyses were performed for the predefined subgroups. Cox proportional hazards models were used to estimate hazard ratios (HRs) for time‐to‐event outcomes, while logistic regression was used where appropriate to estimate odds ratios (ORs). Adjustments were made for sex; history of myocardial infarction; prior PCI; prior CABG; smoking; hypertension; hypercholesterolemia; diabetes mellitus; ACS subtype; lowest hemoglobin level; and highest eGFR, CRP, and troponin levels during admission. In all analyses, values < 1 favored PCI over optimal medical therapy.

## 3 Results

In this 4‐year, all‐comer, consecutive ACS cohort comprising 6168 patients, a total of 775 (13%) octogenarian and nonagenarian patients presented with (N)STEMI and were included in the analysis.

### 3.1 Risk and Profile of the Elderly (N)STEMI Patients

The groups NSTEMI and STEMI comprised 560 and 215 octo and nanogenarian patients, respectively, with median ages of 84 years (interquartile range [IQR] 81–86) and 84 years (IQR 82–87). No significant differences were observed in sex, but cardiac history, diabetes mellitus, or smoking status were more prominent in NSTEMI pts (Table 1). NSTEMI patients were characterized by a predominance of earlier infarction (26%) and previous PCI (31%). Coronary angiography was performed less frequently in NSTEMI than in STEMI (69% vs. 88%; *p* < 0.001). The prevalence of single‐vessel coronary artery disease was less in the NSTEMI versus STEMI group (23% vs. 35%; *p* = 0.001).

**Table 1:** STEMI and NSTEMI in octo and nonagenarians.

	NSTEMI (*N* = 560)	STEMI (*N* = 215)	*p*‐value
Median age [25–75 percentile]	84,0 [81,0–86,0]	84,0 [82,0–87,0]	0,217*
Male (%)	297 (53,0)	121 (56,3)	0,417
Medical history			
Previous MI (%)	146 (26,1)	35 (16,3)	0,004
Previous PCI (%)	176 (31,4)	49 (22,8)	0,018
Previous CABG (%)	46 (8,2)	13 (6,0)	0,346
Risk factors			
Smoking (%)	47 (8,4)	29 (13,5)	0,033
DM (%)	134 (23,9)	33 (15,3)	0,009
Hypertension (%)	380 (67,9)	118 (54,9)	< 0,001
Hypercholesterolemia(%)	223 (39,8)	70 (32,6)	0,062
Laboratory:			
Median Hb at ad. [25–75 percentile]	8,05 [7,30–8,80]	8,20 [7,50–8,70]	0,096*
Median lowest Hb during ad. [25–75 percentile]	7,90 [7,10–8,60]	7,30 [8,10–8,70]	0,076*
Median Hb lost [25–75 percentile]	0,00 [0,00–0,00]	0,00 [0,00–0,00]	0,019*
Median CRP at ad. [25–75 percentile]	3,30 [0,90–12,00]	2,65 [0,90–8,00]	0,316*
Median Max. CRP during ad. [25–75 percentile]	3,80 [1,00–17,00]	3,60 [1,00–14,00]	0,604*
Median highest Troponin during ad. [25–75 percentile]	1558,0 [232,0–8400,0]	28959,0 [7431,3–91486,0]	< 0,001*
Median highest eGFR during ad.	60,0 [46,0–75,0]	63,0 [52,0–78,3]	0,008*
CAG (%)	385 (68,8)	190 (88,4)	< 0,001
PCI (%)	277 (49,5)	182 (84,7)	< 0,001
PCI + CABG (%)	283 (50,5)	182 (84,7)	< 0,001
CABG (%)	6 (1,1)	0 (0,0)	0,195**
CAG:			< 0,001
not significant coronary disease or normal (%)	51 (13,2)	3 (1,6)	+
1 coronary disease (%)	126 (22,5)	75 (34,9)	+
2 coronary disease (%)	97 (17,3)	63 (29,3)	+
3 coronary disease (%)	109 (19,5)	49 (22,8)	+
stopped during CAG	2 (0,5)	0 (0,0)	+
Initial ICU admission (%)	9 (1,6)	4 (1,9)	0,761
Median Admission time (days) [25–75 percentile]	2,43 [1,22–4,89]	2,30 [1,61–3,76]	0,425*
Complications:			
secondary ICU admission (%)	3 (0,5)	1 (0,5)	0,456**
CVA (%)	3 (0,5)	1 (0,5)	1,000**
BARC type 3a (%)	13 (2,7)	5 (2,4)	0,816
Re‐admission with ACS within 365 days (%)	34 (6,1)	9 (4,2)	0,305
In hospital mortality	22 (3,9)	10 (4,7)	0,651
All‐cause death at 30 days (%)	43 (7,7)	25 (11,6)	0,082
All‐cause death at 180 days (%)	77 (13,8)	35 (16,3)	0,370
All‐cause death at 365 days (%)	103 (19,0)	42 (20,0)	0,715

* Mann‐whitney test. ** Fisher exact test.

### 3.2 Biochemistry of the Elderly (NSTEMI Patients

Biochemical parameters reflecting key determinants of frailty and clinical outcomes are summarized below. NSTEMI pts had a lower median peak estimated glomerular filtration rate (eGFR) during admission of 60 mL/min (IQR 46–75). Median hemoglobin (Hb) and CRP levels at admission were comparable between the two groups. The median highest troponin level during admission was significantly higher in the STEMI (28959 ng/l, IQR 7431‐91468 ng/l) versus NSTEMI (1558 ng/l, IQR 232‐8400 ng/l).

### 3.3 Invasive Procedures Versus OMT in (N)STEMI Elderly

Among octogenarian and nonagenarian patients with STEMI, 15% were treated with optimal medical therapy (OMT), whereas 85% (*n* = 182) underwent invasive treatment (Table 2). Cardiovascular risk factors were comparable between the two groups; however, invasively treated patients had a lower prevalence of prior myocardial infarction and prior CABG (Table 2). Patients treated with OMT had significantly lower hemoglobin levels and lower CRP and eGFR values at admission compared with those treated invasively.

**Table 2:** Optimal medical treatment versus invasive treatment.

STEMI			
	OMT *N* = 33	PCI *N* = 182	*P*‐value
Median age [25–75 percentile]	87 [85–90]	83 [82–86]	< 0,001*
Male (%)	18 (54,5)	103 (56,6)	0,827
Previous MI (%)	11 (33,3)	24 (13,2)	0,004
Previous PCI (%)	6 (18,2)	43 (23,6)	0,493
Previous CABG (%)	5 (15,2)	8 (4,4)	0,017
Risk factors			
Smoking (%)	2 (6,1)	27 (14,8)	0,175
DM (%)	5 (15,2)	28 (15,4)	0,973
Hypertension (%)	19 (57,6)	99 (54,4)	0,736
Hypercholesterolemia (%)	8 (24,2)	62 (34,1)	0,268
Laboratory:			
Median Hb at ad. [25–75 percentile]	7,7 [7,20–8,50]	8,2 [7,50–8,90	0,045*
Median lowest Hb during ad. [25–75 percentile]	7,70 [7,10–8,50]	8,10 [7,30–8,80]	0,118*
Median Hb lost [25–75 percentile].	0,00 [0,00–0,00]	0,00 [0,00–0,00]	0,640*
Median CRP at ad. [25–75 percentile]	8,05 [0,68–64,25]	2,40 [0,90–6,88]	0,019*
Median Max. CRP during ad. [25–75 percentile]	11,15 [0,93–77,25]	3,10 [0,98–11,00]	0,030*
Median highest Troponin during ad. [25–75 percentile]	9301,50 [2675,75‐28915,50]	40003,50 [9176,00‐101298,25]	< 0,001
Median highest eGFR during ad. [25–75 percentile]	57,00 [45,00–70,00]	64,00 [53,00–79,00]	0,018
CAG (%)	8 (24,2)	182 (100)	< 0,001
CABG (%)	*	0 (0,0)	1
PCI (%)	*	181 (99%)	< 0.001
not significant coronary disease or normal	2 (25,0)	0 (0)	*
1 coronary disease (%)	3 (37,5)	73 (40,1)	*
2 coronary disease (%)	1 (12,5)	62 (34,1)	*
3 coronary disease (%)	2 (25,0)	47 (25,8)	*
stopped during CAG	0 (0,0)	0 (0,0)	*
Initial ICU admission (%)	0 (0,0)	4 (2,2)	1,00**
Complications:			
secondary ICU admission	0,0 (0,0)	1 (0,5)	1,00**
CVA	0 (0,0)	1 (0,5)	1,00**
BARC type 3a (%)	1 (3,2)	4 (2,2)	0,554**
Re‐admission with ACS	3 (9,1)	6 (3,3)	0,144**
In‐Hospital mortality (%)	3 (9,1)	7 (3,8)	0,185**
All‐cause death at 30 days (%)	8 (24,2)	17 (9,3)	0,033
All‐cause death at 180 days (%)	11 (33,3)	24 (13,2)	0,004
All‐cause death at 365 days (%)	12 (36,4)	30 (16,5)	0,008

NSTEMI			
	OMT *N* = 277	PCI + CABG *N* = 283	*P*‐value
Median age [25–75 percentile]	85 [82–88]	83 [81–85]	< 0,001*
Male (%)	129 (46,6)	168 (59,4)	0,002
Previous MI (%)	81 (29,2)	65 (23,0)	0,091
Previous PCI (%)	83 (30,0)	93 (32,9)	0,460
Previous CABG (%)	31 (11,2)	15 (5,3)	0,011
Risk factors			
Smoking (%)	28 (10,1)	19 (6,7)	0,147
DM (%)	60 (21,7)	74 (26,1)	0,213
Hypertension (%)	195 (70,4)	185 (65,4)	0,203
Hypercholesterolemia (%)	117 (42,2)	106 (37,5)	0,248
Laboratory:			
Median Hb at ad. [25–75 percentile]	7,80 [6,90–8,50]	7,70 [8,30–9,00]	< 0,001*
Median lowest Hb during ad. [25–75 percentile]	7,70 [6,70–8,30]	8,20 [7,60–8,80]	< 0,001*
Median Hb lost [25–75 percentile]	0,00 [0,00–0,00]	0,00 [0,00–0,00]	0,501*
Median CRP at ad. [25–75 percentile]	5,20 [1,30–18,00]	2,050 [0,70–7,18]	< 0,001*
Median Max. CRP during ad. [25–75 percentile]	6,20 [1,40–43,00]	2,30 [0,73–9,18]	< 0,001*
Median highest Troponin during ad. [25–75 percentile]	1135,50 [135,50–5632,00]	2449,00 [393,00–12255,00]	< 0,001*
Median highest eGFR during ad. [25–75 percentile]	56,00 [43,00–74,00]	62,00 [51,00–75,50]	0,005*
CAG (%)	102 (36,8)	283 (100)	< 0,001
CABG	0	6 (2,1)	0,030**
PCI (%)	0	277 (97,9)	< 0,001
not significant coronary disease or normal	50 (49,0)	0 (0,0)	*
1 coronary disease (%)	15 (14,7)	112 (39,2)	*
2 coronary disease (%)	12 (11,8)	85 (30,0)	*
3 coronary disease (%)	23 (22,5)	86 (30,4)	*
stopped during CAG	2 (2,0)	0 (0,0)	*
Initial ICU admission (%)	8 (2,9)	1 (0,4)	0,019
Complications:			
secondary ICU admission	3 (1,1)	0 (0,0)	0,120**
CVA	2 (0,7)	1 (0,4)	0,620**
BARC type 3a (%)	8 (3,1)	5 (2,2)	0,779**
Re‐admission with ACS	19 (6,9)	15 (5,3)	0,482**
In‐Hospital mortality	16 (5,8)	6 (2,1)	0,030
All‐cause death at 30 days (%)	33 (11,9)	10 (3,5)	< 0,001
All‐cause death at 180 days (%)	59 (21,3)	18 (6,4)	< 0,001
All‐cause death at 365 days (%)	79 (28,8)	24 (8,5)	< 0,001

* Mann‐whitney test. ** Fisher exact test.

Coronary angiography was performed in 24% of patients managed with OMT, without subsequent PCI or CABG, and in all patients treated invasively. Among invasively treated patients, multivessel coronary artery disease was present in 60%.

Among STEMI patients undergoing coronary angiography, primary PCI was performed in 181 of 182 cases, with multivessel PCI in 8; procedural success was 98%. No patients underwent CABG.

Among patients with NSTEMI, 283 (51%) octogenarian and nonagenarian patients were treated with an invasive strategy. Cardiovascular risk factors were comparable between groups; however, invasively treated patients less frequently had a history of CABG (Table 2). Patients managed with optimal medical therapy (OMT) had significantly higher hemoglobin and CRP levels, as well as lower eGFR and troponin levels during admission, compared with invasively treated patients.

Coronary angiography was performed in 37% of patients managed with OMT and in all invasively treated patients. Multivessel coronary artery disease was identified in 40% of OMT patients and 60% of invasively treated patients. PCI was subsequently performed in 277 NSTEMI patients, with a procedural success rate of 98%. CABG was performed in six patients with extensive multivessel disease. Heart team meetings' decisions to refrain from interventions were based upon the clinical angina score, frailty, and/or too extensive coronary artery disease.

### 3.4 Clinical Outcomes in (N)STEMI Elderly

#### 3.4.1 STEMI

In‐hospital complications did not differ between treatment groups (Table 2). BARC type 3a bleeding occurred in one patient in the OMT group and four patients in the invasive group (*p* = 0.55). In‐hospital cerebrovascular events were rare and occurred in one invasively treated STEMI patient. In‐hospital mortality was comparable between patients treated with OMT and those treated invasively (*n* = 3 vs. *n* = 7; *p* = 0.19). Similarly, readmission rates for recurrent ACS within 1 year did not differ significantly between the OMT and invasive groups (*n* = 3 vs. *n* = 6; *p* = 0.144).

#### 3.4.2 NSTEMI

In‐hospital complications did not differ between treatment groups (Table 2). BARC type 3a bleeding occurred in 8 of invasively treated patients and 5 patients treated with optimal medical therapy (OMT) (*p* = 0.78). In‐hospital cerebrovascular events were rare, occurring in 2 of the invasively treated patients and 1 of the OMT‐treated patients (*p* = 0.62). Readmission rates for recurrent ACS < 1 year were low and comparable between groups (*n* = 19 OMT vs. *n* = 15 invasive group). In‐hospital mortality was significantly lower in invasively treated patients compared with those managed with OMT (*n* = 16 vs *n* = 6; *p* = 0.03).

### 3.5 Long‐Term Mortality

**Figure 1:**
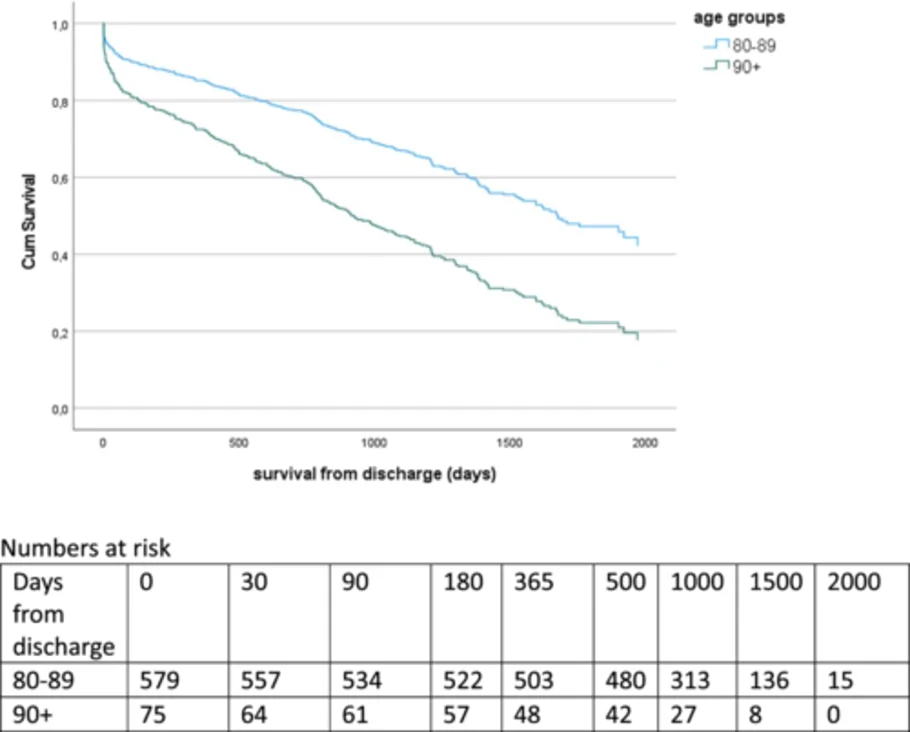
Survival analysis for (N)STEMI octo and nonagenarian patients. Crude: 2757 [2094–3630] < 0,001. Corrected: 2028 [1512–2718] *p* = < 0,001. 2007 [1498–2690] *p* = < 0,001. Corrected for: gender, MI, CABG, PCI, DM, smoking, hypertension, hypercholestrolemia, e‐GFR highest, tropo highest, CRP highest, Hb lowest, Diagnose: NSTEMI or STEMI, treatment (OMT or PCI/CABG), 365 Mortality regression analysis: Crude: 3154 [1931–5154] *p* = < 0,001. Corrected: 2259 [1280–3987] *p* = 0,005.

**Figure 2:**
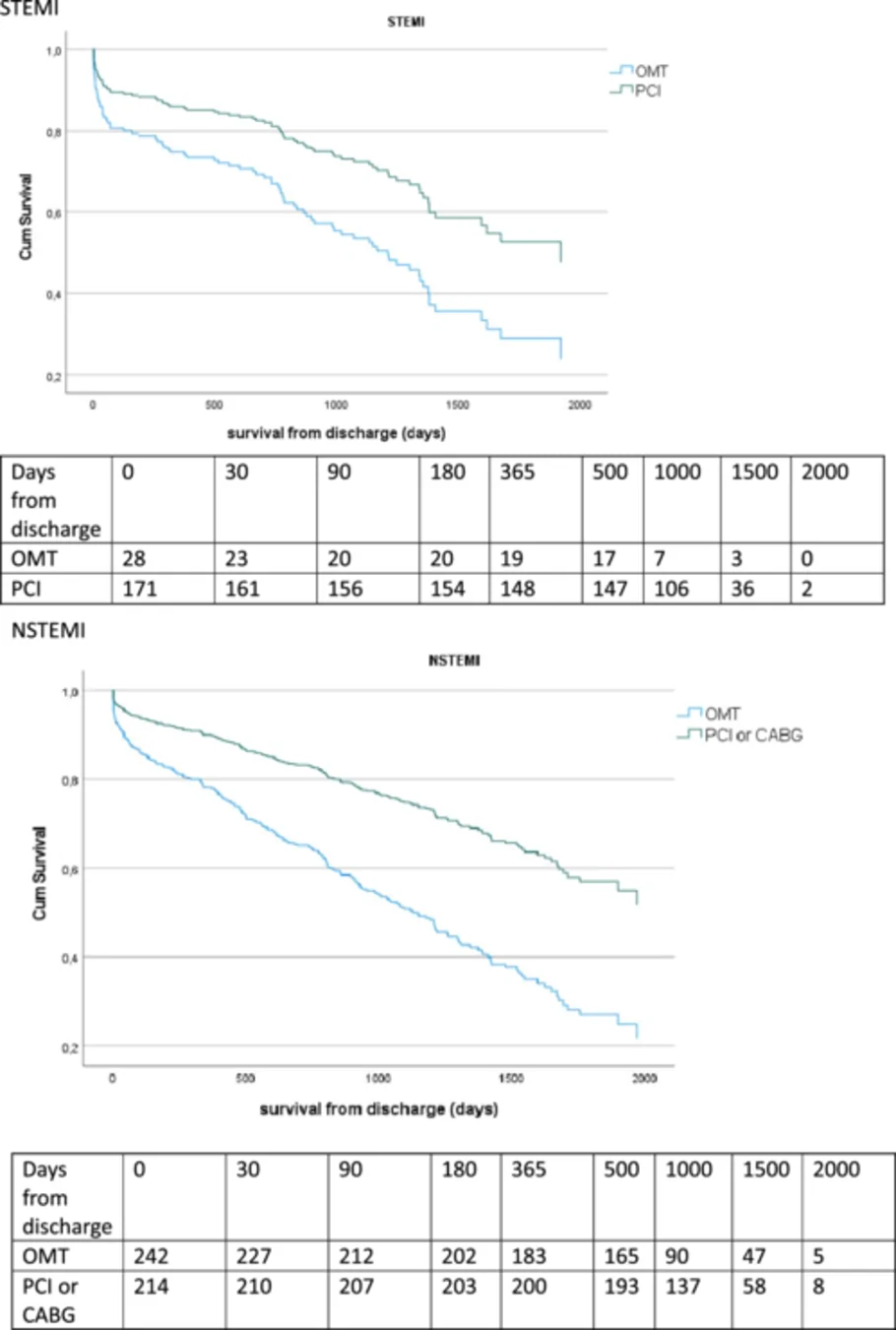
Survival analysis for invasive and conservative therapy in (N)STEMI octo and nonagenarian patients. Crude: 0,314 [0,197–0,500] *p* = < 0,001. Corrected: 0,518 [0,267–1,006] *p* = 0,052. Corrected for: gender, MI, CABG, PCI, DM, smoking, hypertension, hypercholestrolemia, e‐GFR highest, tropo highest, CRP highest, Hb lowest, age in decimals. 365 Mortaliteit regression analysis: Crude: 0,345 [0,154–0,777] *p* = 0,010. Corrected: 0,725 [0,225–2,339] *p* = 0,591. Crude: 0,390 [0,301–0,506] *p* = < 0,001. Corrected: 0,430 [0,315–0,585] *p* = < 0,001. Corrected for gender, MI, CABG, PCI, DM, smoking, hypertension, hypercholestrolemia, e‐GFR highest, tropo highest, CRP highest, Hb lowest, age in decimals. Corrected analyses: OMT: 258, PCI/CABG: 219. 365 Mortaliteit regressieanalyse: Crude: 0,232 [0,142–0,380] *p* = < 0,001. Corrected: 0,258 [0,139–0,478] *p* = < 0,001.

All patients were followed for a median of 999 days (IQR 602–1420). At 30 days, mortality differed significantly between age groups, with rates of 7.8% in patients aged 80–89 years and 17% in those aged ≥ 90 years (*p* = 0.011) (Figure 1). This difference persisted at 180 days (13% vs. 26%; *p* < 0.004) and 365 days (17% vs. 39%; *p* < 0.001) (Figure 2). After adjustment for relevant covariates, the odds of 365‐day mortality were significantly higher in patients aged ≥ 90 years compared with those aged 80–89 years (adjusted odds ratio 2.26, 95% CI 1.28–3.99; *p* = 0.005).

In the STEMI cohort, 1‐year mortality was significantly lower in invasively treated patients compared with those managed with optimal medical therapy (*n* = 30 (16.5%) vs. *n* = 12 (36.4%); *p* = 0.008) (Figure 2). Among patients with NSTEMI, mortality at 1 year was markedly lower in invasively treated patients than in those managed with OMT (*n* = 24 (8.5%) vs. *n* = 79 (29%); *p* < 0.001). When stratified by ACS subtype, adjusted hazard ratios for mortality favored invasive treatment only in the NSTEMI group. In the STEMI group, the adjusted hazard ratio was 0.52 (*p* = 0.052), whereas in the NSTEMI group, the adjusted hazard ratio was 0.430 (*p* < 0.001).

### 3.6 Sex‐Related Differences in Elderly (N)STEMI Patients

Cardiovascular risk profiles and clinical outcomes differed modestly between female and male octogenarian and nonagenarian patients (Table 3). Women were, on average, slightly older than men (84 vs. 83 years), had a lower prevalence of prior myocardial infarction (27% vs. 19%; *p* = 0.009), and presented with lower hemoglobin levels. The distribution of ACS subtype was similar between sexes, with no significant differences in NSTEMI (72%) and STEMI (28%) presentation or in troponin levels.

**Table 3:** Clinical characteristics of male and female (N)STEMI octo and nonagenarians.

	Total population (*n* = 775)	Male (*n* = 418)	Female (*n* = 357)	*p*‐value
Median age [25–75 percentile]	84,0 [82,0–87,0]	83 [82–86]	84 [82–87]	0,031*
Male (%)	418 (53,9)	418 (100)	0 (0,0)	x
Medical History				
Previous MI (%)	181 (23,4)	113 (27,0)	68 (19,0)	0,009
Previous PCI (%)	225 (29,0)	129 (30,9)	96 (26,9)	0,225
Previous CABG (%)	59 (7,6)	42 (10,0)	17 (4,8)	0,006
Risk factors				
Smoking (%)	76 (9,8)	38 (9,1)	38 (10,6)	0,469
DM (%)	167 (21,5)	91 (21,8)	76 (21,3)	0,871
Hypertension (%)	498 (64,3)	259 (62,0)	239 (66,9)	0,149
Hypercholesterolemia (%)	293 (37,8)	156 (37,3)	137 (38,4)	0,763
ACS Type				
STEMI (%)	215 (27,7)	121 (28,9)	94 (26,3)	0,417
NSTEMI (%)	560 (72,3)	297 (71,1)	263 (73,7)	0,417
Laboratory:				
Median Hb at ad. [25–75 percentile]	8,10 [7,30–8,73]	8,30 [7,60–9,00]	7,90 [7,20–8,50]	< 0,001
Median lowest Hb during ad. [25–75 percentile]	8,00 [7,10–8,60]	8,20 [7,30–8,80]	7,80 [7,00–8,40]	< 0,001
Median Hb lost [25–75 percentile]	0,00 [0,00–0,00]	0,00 [0,00–0,00]	0,00 [0,00–0,00]	0,820
Median CRP at ad. [25–75 percentile]	3,10 [0,90–11,0]	3,30 [0,90–14,00]	3,10 [0,95–8,45]	0,273
Median Max. CRP during ad. [25–75 percentile]	3,80 [1,00–17,00]	4,20 [1,00–‐23,50]	3,40 [1,00–11,00]	0,211
Median highest Troponin during ad. [25–75 percentile]	4039,0 [420,0–21280,0]	4484,0 [494,5–23141,3]	3849,0 [343,0–20541,0]	0,223
Median highest eGFR during ad.	61,0 [48,0–76,0]	60,0 [48,0–75,0]	63,0 [48,0–77,5]	0,251
CAG (%)	575 (74,2)	324 (77,5)	251 (70,3)	0,022
PCI (%)	459 (59,2)	266 (63,6)	193 (54,1)	0,007
PCI + CABG (%)	465 (60,0)	271 (64,8)	194 (54,3)	0,003
CABG (%)	6 (0,8)	5 (1,2)	1 (0,3)	0,147
not significant coronary disease or normal (%)	52 (9,1)	20 (6,1)	32 (12,8)	0,003
1 coronary disease (%)	203 (35,3)	105 (32,4)	98 (39,0)	0,003
2 coronary disease (%)	160 (27,8)	92 (28,4)	68 (27,1)	0,003
3 coronary disease (%)	158 (27,5)	106 (32,7)	52 (20,7)	0,003
stopped during CAG	2 (0,3)	1 (0,3)	1 (0,4)	0,004
Initial ICU admission (%)	11 (1,7)	6 (1,4)	7 (2,0)	0,570
Median Admission time (days) [25–75 percentile]	2,39 [1,25–4,29]	2,49 [1,45–4,94]	2,25 [1,03–3,94]	0,102*
Complications:				
secondary ICU admission (%)	4 (0,5)	1 (0,2)	3 (0,8)	0,244
CVA (%)	4 (0,5)	4 (1,0)	0 (0,0)	129**
BARC type 3a (%)	18 (2,6)	7 (1,8)	11 (3,5)	0,175
Re‐admission with ACS (%)	43 (5,5)	21 (5,0)	22 (6,2)	0,490
In hospital mortality	32 (4,1)	24 (5,7)	8 (2,2)	0,015
All‐cause death at 30 days (%)	68 (8,8)	41 (9,8)	27 (7,6)	0,271
All‐cause death at 180 days (%)	112 (14,5)	65 (15,6)	47 (13,2)	0,347
All‐cause death at 365 days (%)	145 (18,7)	87 (20,8)	58 (16,2)	0,104
NSTEMI All‐cause death at 365 days (%)	103 (18,4)	61 (20,5)	42 (16,0)	0,164
STEMI All‐cause death at 365 days (%)	42 (19,5)	26 (21,5)	16 (17,0)	0,413

* Mann‐whitney test. ** Fisher exact test.

**Figure 3:**
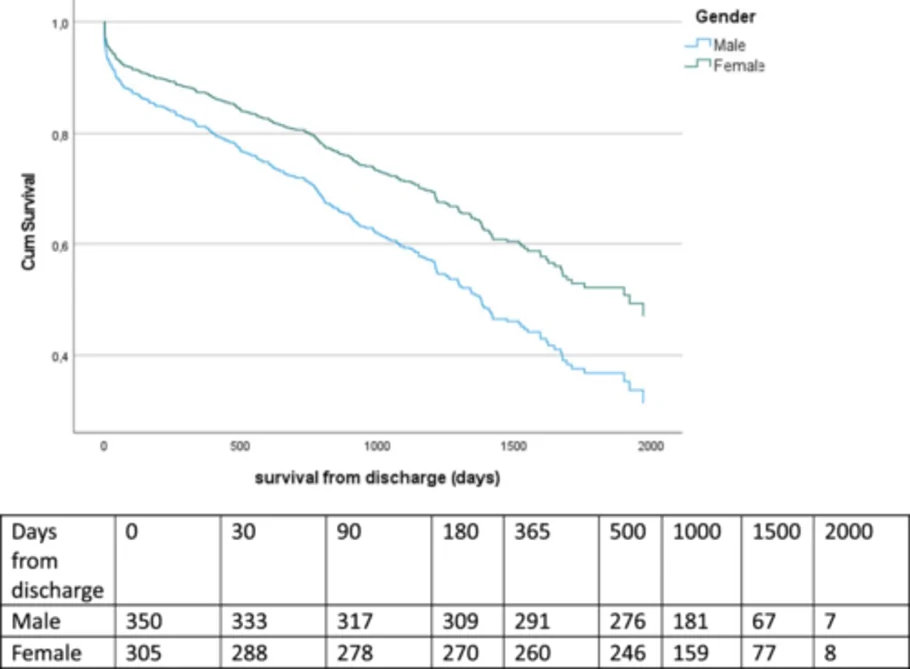
Survival analysis for male versus female (N)STEMI octo and nonagenarian patients. Crude survival analysis: 0,795 (0,641–0,985) *p* = 0,036. Corrected Cox‐survival analysis HR: 0,651 [0,513–0,825] *p* = < 0,001. Corrected for age decimals, VG: MI, PCI, CABG, DM, hypercholesterolemia, hypertension, smoking, ACS type, Hb lowest, eGFR highest, CRP highest, tropo highest, and treatment (PCI/CABG). 365 Mortality regression analysis: Crude: 0,738 [0,511–1,065] *p* = 0,105. Corrected: 0,556 [0,360‐0,860] *p* = 0,008.

Despite comparable ACS characteristics, coronary angiography (70% vs. 78%; *p* = 0.02) and PCI (54% vs. 65%; *p* = 0.003) were performed significantly less frequently in women than in men. This difference was accompanied by a higher prevalence of non‐obstructive coronary artery disease in women (13% vs. 6%; *p* = 0.003). At 365 days of follow‐up (Figure 3), no significant difference in all‐cause mortality was observed between women and men (16% vs. 21%; *p* = 0.10).

## 4 Discussion

Over a 4‐year period, a regional high‐volume PCI center treated 6168 consecutive all‐comer ACS patients, of whom 775 (13%) were octo or nonagenarians. Nonagenarians exhibited a distinct risk profile compared with octogenarians, characterized by a predominance of women, a higher prevalence of hypertension, and a lower prevalence of hypercholesterolemia. Although coronary angiography and PCI were performed less frequently in nonagenarians, invasive management was associated with superior outcomes compared with medical therapy alone across both age groups. Among elderly postmenopausal women with (N)STEMI, coronary artery disease was less obstructive, and invasive strategies were used less often; nevertheless, long‐term outcomes were comparable to those of elderly men.

Octogenarians and nonagenarians in Europe have a substantial remaining life expectancy, averaging approximately 6 and 3 years for men aged 85 and 95 years, and 7 and 3 years for women, respectively [12, 13]. This suggests that very elderly patients presenting with (N)STEMI may be fitter than often assumed, potentially reflecting declining smoking prevalence over recent decades [13]. These considerations are highly relevant when weighing invasive treatment strategies in this population.

In the present study, octogenarian and nonagenarian patients with (N)STEMI demonstrated a relatively favorable prognosis, particularly when treated invasively. At a median follow‐up of 999 days, survival rates among invasively treated patients were 83% for STEMI and 91% for NSTEMI. In contrast, patients managed with optimal medical therapy had lower survival rates, likely reflecting greater frailty, comorbidity burden, and higher baseline risk at presentation rather than a treatment effect alone. Importantly, complication rates associated with invasive management were low, even in very elderly patients. Across both STEMI and NSTEMI, invasive treatment was associated with superior long‐term outcomes compared with medical therapy, underscoring that benefit is achievable with appropriate patient selection.

These findings are consistent with prior observational studies in elderly NSTEMI populations, which have reported improved all‐cause survival with PCI [14, 15, 16, 17]. Although the observational design precludes exclusion of selection bias, our data indicate that PCI in very elderly NSTEMI patients does not confer excess procedural risk and can be performed safely in carefully selected individuals.

In STEMI patients, invasive treatment was associated with improved crude outcomes; however, this effect did not remain statistically significant after adjustment, likely due to the small number of conservatively treated patients. Nevertheless, the direction of effect favored PCI and aligns with evidence from randomized trials in patients aged ≥ 75 years, which demonstrated reduced mortality rate with invasive management [18, 19]. In addition, pooled analyses from the TRIANA trial reported improved composite outcomes—including death, reinfarction, and disabling stroke—in patients treated with PCI [4].

### 4.1 Patient Selection

Managing acute coronary syndromes in octo and nonagenarian patients requires careful patient selection, particularly in STEMI, where commonly used risk scores lack validation and applicability in the acute setting.

Our real‐world data suggest that in acute STEMI, clinical parameters such as cognitive status, functional capacity, and pre‐existing left ventricular dysfunction or heart failure may help identify very elderly patients who are most likely to benefit from primary PCI. In NSTEMI, the longer diagnostic window allows for broader clinical evaluation, including geriatric assessment when needed. Accordingly, our findings indicate that invasive management can be appropriately selected in very elderly patients, with the greatest benefit observed in NSTEMI patients aged ≥ 90 years.

Decisions regarding PCI in the very elderly should therefore be individualized and guided by shared decision‐making, taking into account patient preferences, life expectancy, comorbidity burden, ACS subtype, and troponin levels.

### 4.2 Gender Differences in the Very Elderly

To assess sex‐related differences in very elderly patients with (N)STEMI, we compared outcomes between women and men. Women were older, had fewer prior myocardial infarctions and revascularization procedures, and less obstructive coronary artery disease, while traditional risk factors were largely similar. Despite less frequent use of coronary angiography and PCI, long‐term outcomes in women were comparable to those in men.

Sex‐specific differences in cardiovascular biology, particularly after menopause, contribute to increased cardiovascular risk in women later in life [20, 21, 22]. Although coronary artery disease typically presents at an older age in women, evidence indicates that invasive management remains effective in elderly female patients. Prior studies have shown that PCI in very elderly women with ACS reduces non‐fatal myocardial infarction and cardiovascular mortality without excess procedural risk, supporting invasive strategies in carefully selected patients.

### 4.3 Limitations

This study has several limitations. Due to its observational design, residual confounding and selection bias cannot be excluded, despite the use of multivariable adjustment. In addition, clinical characteristics and diagnoses were derived from physician‐reported data in the electronic health record, which may be subject to misclassification, and we chose the variables used in the Dutch and EuroHeart Registries [23, 24]. These do not include ejection fraction or peripheral arterial disease.

Additionally, in line with the registries, we consider all‐cause mortality a more unequivocal primary endpoint than cardiovascular death for several reasons. First, all deaths are included, given that the municipality checks for all patients; secondly, the cause of death in these elderly patients is difficult to determine, given the multiple chronic diseases. An additional analysis of the data showed that in the last 60 days before death, 3 (0.9%) patients suffered a STEMI or NSTEMI, 11 (3%) had an ischemic CVA or TIA, and 8 (2.3%) patients had an oncology intervention. Finally, many patients were returned to the care of their family doctor at their 1‐year follow‐up date. At long‐term follow‐up (> 12 months), the median time between the last cardiology visit and death was 323 (IQR 0‐1801) days.

Nevertheless, this study provides valuable real‐world evidence in octogenarian and nonagenarian patients, a population for which randomized clinical data remain scarce.

## 5 Conclusion

In this real‐world cohort of very elderly patients with ACS, appropriately stratified octo and nonagenarians benefited from invasive management without excess complications, with the greatest benefit observed in NSTEMI patients aged ≥ 90 years. This supports an individualized, patient‐centered approach to invasive treatment decisions.

## Funding

The authors have nothing to report.

## Data Availability

The data that support the findings of this study are available on request from the corresponding author. The data are not publicly available due to privacy or ethical restrictions.
